# Impact of Chronic Levels of Naturally Multi-Contaminated Feed with *Fusarium* Mycotoxins on Broiler Chickens and Evaluation of the Mitigation Properties of Different Titers of Yeast Cell Wall Extract

**DOI:** 10.3390/toxins12100636

**Published:** 2020-10-01

**Authors:** Alexandra C. Weaver, W. D. King, Morgan Verax, Ursula Fox, Manoj B. Kudupoje, Greg Mathis, Brett Lumpkins, Alexandros Yiannikouris

**Affiliations:** 1 Alltech, Inc., 3031 Catnip Hill Road, Nicholasville, KY 40356, USA; aweaver@alltech.com (A.C.W.); wdking@alltech.com (W.D.K.); mverax@alltech.com (M.V.); ufox@alltech.com (U.F.); mkudupoje@alltech.com (M.B.K.); 2 Southern Poultry Research, Inc., Athens, GA 30607, USA; southern_poultry_res@msn.com (G.M.); southernpoultry@gmail.com (B.L.)

**Keywords:** broiler, gastrointestinal, mycotoxins, mycotoxin mitigation, yeast cell wall extract

## Abstract

The chronic intake of naturally multi-mycotoxin contaminated feed by broilers with or without titers of Yeast Cell Wall Extract (YCWE, a.k.a Mycosorb A+^®^), was investigated. Day-old male Cobb chicks (1600 birds, 64 pens, 25 birds/pen) were randomly allocated to diets of control (CON); diet containing mycotoxins (MT); CON + 0.2% YCWE; MT + 0.025% YCWE; MT + 0.05% YCWE; MT + 0.1% YCWE; MT + 0.2% YCWE; and MT + 0.4% YCWE. Growth performance, blood biochemical parameters and gut health were recorded over 42 days. Compared with CON, MT had reduced body weight (BW) and increased feed conversion ratio (FCR) on days 35 and 42 with increased duodenal crypt depth and fewer goblet cells. Furthermore, European Poultry Production Efficiency (EPEF) was reduced for MT versus CON. Feeding MT + 0.2% YCWE improved BW, lowered FCR, reduced crypt depth, increased goblet cell count and improved EPEF. Considering titration of YCWE (0 to 0.4%) during mycotoxin challenge, a cubic effect was observed for FCR with NC + 0.2% YCWE having the lowest FCR. These findings suggest that chronic consumption of multiple *Fusarium* mycotoxins present in common field concentrations can negatively impact broiler performance and gut health while inclusion of YCWE, particularly 0.2%, could be effective in counteracting mycotoxins.

## 1. Introduction

Globally, mycotoxins are a common natural contaminant of feedstuffs and feeds [1]. There are hundreds of mycotoxins that can impact poultry with some of the most important being aflatoxin B1 (AFB1), ochratoxin A, deoxynivalenol (DON), T-2/HT-2 toxins, zearalenone, fumonisins, moniliformin and cyclopiazonic acid [2,3,4]. Although mycotoxins have diverse biological and toxicological activities in animals, many of them do lower growth performance, cause damage to internal organs, alter immunity and increase disease susceptibility. The United States Food and Drug Administration (FDA) and European Food Safety Authority (EFSA) have regulatory guidance for poultry feed for only few mycotoxins including DON and fumonisin [5], AFB1 being the only mycotoxin with regulatory limits. Although intake of individual mycotoxins at acute levels above regulations can occur, chronic intake of lower to moderate concentrations of mycotoxins is likely more common. Exposure to the latter may play an important role in broiler performance and health when consumed from the starter through finisher phases [6]. Furthermore, the consumption of mixtures of mycotoxins can lead to an increase in toxicological response [7]. The natural co-occurrence of mycotoxins into feeds ensues several possible reasons that could all be intertwined. These include the ability of molds to produce a plurality of mycotoxins; a single commodity can contain multiple molds that produce multiple mycotoxins, and finally complete rations usually combine several feed ingredients that can each contain unique, similar or different types of mycotoxins [8]. The toxicity of mixtures of mycotoxins cannot always be predicted, but mycotoxin combination can lead to additive, synergistic or even antagonistic interactions [9].

As mycotoxins in feedstuffs may not be avoided, one of the most practical methods to reduce the risk of mycotoxins to poultry is the use of effective mitigation programs that can limit the bioavailability of mycotoxins in the digestive tract of the animal. As such, adsorbents are often supplemented to animal diets. Depending on the nature of the mitigation strategy used, materials could enter either the feed ingredients (such as yeast cell wall extract (YCWE)) or feed additive (i.e., bentonites) categories, the latter fitting into a mycotoxin detoxification/adsorbent category, which varies according to the country, and available legislation. In this context, YCWE has been researched for its ability to adsorb mycotoxins and create an adsorbent–toxin complex. The function of this adsorbent is to bind and maintain the mycotoxin within the digestive tract of the animal, thereby transporting that toxin through the body without exposing internal organs to the harmful effects of mycotoxins [10]. Furthermore, for an adsorbent to be effective its function should not be altered by the digestion properties of the animal, such as pH. Yeast cell wall extract has been investigated as an organic adsorbent shown to sequester numerous mycotoxins, individually or in combination, both in vitro and in vivo [4,10,11]. However, few trials have investigated the ability of a variety of YCWE inclusion rates to alleviate a chronic challenge of multiple mycotoxins at doses that do not exceed the guidance of FDA or EFSA for commercial broilers feeds. Thus, the present work hypothesized that YCWE might mitigate the adverse effects of mycotoxins on performance and gut integrity of broilers chronically consuming mycotoxins. The objectives were to evaluate the impact of chronic feeding of naturally multi-mycotoxin feed on performance, blood metabolites and gut integrity of broiler chickens and to evaluate the impact of mitigation strategy using titrated levels of YCWE containing an algal component (Mycosorb™ A+, Alltech, KY, USA) in a large scale poultry production environment.

## 2. Results

### 2.1. Dietary Mycotoxin Concentrations

Dietary concentrations of mycotoxins are given in Table 1. The level of inclusion of naturally contaminated distillers dried grains with solubles (DDGS) was formulated to target similar mycotoxin concentrations across treatments containing mycotoxins. However, mycotoxin type and concentration did vary between treatments. Deoxynivalenol concentration was lowest in the control (CON) and CON + 0.2% YCWE diets (119.8 and 695.4 µg/kg, respectively), although these two treatments still differed greatly because of the presence of background levels of toxins. The DON content was highest in mycotoxins (MT) diet (2263.8 µg/kg). Diets containing mycotoxins with YCWE varied in their DON content from 1264.0 µg/kg (MT + 0.025% YCWE) to 1937.2 µg/kg (MT + 0.4% YCWE). The HT-2 toxin was detected in three treatments at a range of 95.0 to 165.2 µg/kg. The HT-2 toxin was also detected in the basal diet. All treatments except MT contained some amount of fumonisins (fumonisins B1, B2 or B3).

### 2.2. Statistical Analysis 1

#### 2.2.1. Performance

Mortality rates did not differ between treatments during this trial (data not shown). During the first 28 days, body weight did not differ between treatments (Table 2). However, by day 35, broilers consuming MT had reduced (*p* < 0.001) body weight compared with birds fed CON by 8.33%. On day 42, MT fed birds continued to have lower (*p* < 0.001) body weight in contrast to CON by 8.96%. Broilers fed MT + 0.2% YCWE had increased (*p* = 0.020) body weight compared with MT. Birds consuming CON + 0.2% YCWE or MT + 0.2% YCWE did not differ from birds fed CON by day 42. 

Feed intake of chicks during the first 7 days was reduced (*p* = 0.027) by 19.4% when consuming MT compared with CON (Table 2). On day 14, feed intake was not impacted by treatment. On day 21, consumption of mycotoxins alone in MT reduced (*p* = 0.003) feed intake of broilers compared with CON. Birds fed CON + 0.2% YCWE had greater feed intake (*p* = 0.022) than CON fed birds on day 21. On day 28, there was no impact of mycotoxins on feed intake, but birds fed CON + 0.2% YCWE had greater intake than CON. By day 35 and 42, broilers fed MT had reduced feed intake (*p* < 0.05) compared with CON while those fed CON + 0.2% YCWE had increased feed intake in contrast to CON. Feed intake of MT + 0.2% YCWE did not differ from MT for any week, however these birds did have lower feed intake than CON on days 7 and 28. For the other weeks, feed intake of MT + 0.2% YCWE did not differ from CON.

The feed conversion ratio (FCR) of chicks during the first 7 days was lower for those consuming MT (*p* = 0.004), MT + 0.2% YCWE (*p* = 0.005) and CON + 0.2% YCWE (*p* = 0.030) in contrast to CON (Table 2). On days 14, 21 and 28 there were no significant differences between CON, MT and 0.2% YCWE treatments. However, on days 35 and 42, broilers fed MT had increased (*p* < 0.001) FCR compared with CON. Broilers fed MT + 0.2% YCWE had lower (*p* < 0.05) FCR than MT. The FCR value of CON + 0.2% YCWE and MT + 0.2% YCWE did not differ from CON during these last two weeks.

#### 2.2.2. Blood Parameters

On day 21, adenosine deaminase (ADA) was increased (*p* = 0.008) in blood samples from birds fed MT in contrast to CON, whereas the birds fed MT + 0.2% YCWE had lower (*p* = 0.032) blood ADA than MT (Table 3). Broilers fed CON + 0.2% YCWE had higher ADA compared with both CON (*p* = 0.004) and MT + 0.2% YCWE (*p* = 0.014). Free cholesterol (FCHOL) did not differ between CON and MT, but broilers fed MT + 0.2% YCWE had lower FCHOL than MT and CON. Antioxidant biomarker glucose 6-phosphate dehydrogenase (G6PDH) was restored in MT + 0.2% YCWE (*p* < 0.05) compared to MT. Other blood parameters and markers of liver dysfunction such as aspartate aminotransferase, alanine aminotransferase, apolipoprotein B and lecithin cholesterol acyltransferase (LCAT) were not impacted. On day 42, there were no treatment differences for any blood parameters measured (Table 3).

#### 2.2.3. Intestinal Morphology

**Figure 1:**
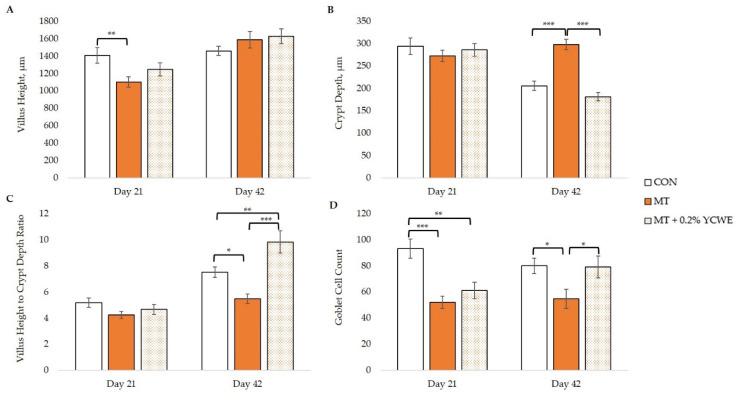
Effect of a natural multi-mycotoxin challenge on duodenal morphology of villus height (**A**), crypt depth (**B**), villus height to crypt depth ratio (**C**) and goblet cell count (**D**) for broilers fed with or without a yeast cell wall extract (YCWE) (mean ± SE). CON: control diet with minimal mycotoxins; MT: diet containing mycotoxin challenge; MT + 0.2% YCWE. Significant contrasts between treatments were noted (* *p*-value < 0.05; ** *p*-value < 0.01; *** *p*-value < 0.001).

**Figure 2:**
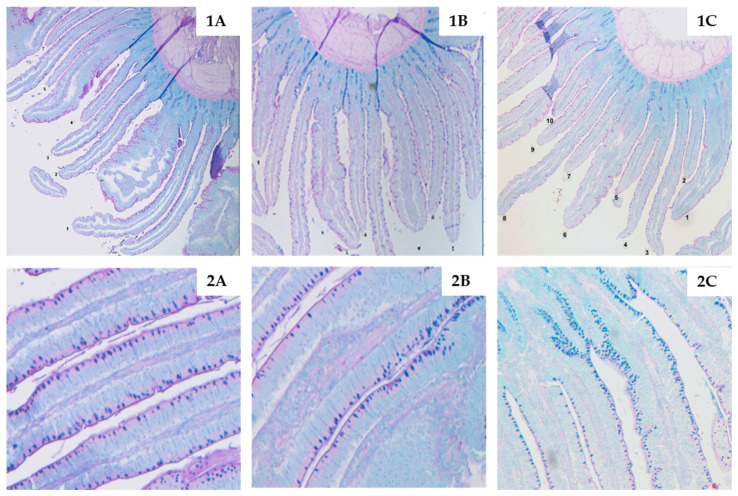
Histological observations of the villi using light microscopy after staining and processing of duodenum samples collected on day 42 for broilers fed (**A**) control diet with minimal background levels of mycotoxins (CON); (**B**) a diet containing a mycotoxin challenge (MT); or (**C**) MT + 0.2% yeast cell wall extract (YCWE). Compared with the duodenum of birds fed CON, crypt depth was increased in the duodenum of birds fed MT which was again reduced in birds fed MT + 0.2% YCWE (images **1A**, **1B** and **1C**). Goblet cell count was reduced in the duodenum of birds fed MT compared with those fed CON or MT + 0.2% YCWE (images **2A**, **2B** and **2C**).

Analysis of the duodenum on day 21 (Figure 1) showed that broilers consuming MT had reduced duodenal villi height (*p* = 0.005) and reduced goblet cell count (*p* < 0.001) compared with birds fed CON. Broilers fed MT + 0.2% YCWE did not differ from either CON or MT for the duodenal morphology parameters measured. Villi height and goblet cell count for the duodenum of birds fed MT + 0.2% YCWE were increased compared with those in MT (*p* = 0.002 and 0.006, respectively). On day 42 (Figure 1 and Figure 2), villi height was not impacted by treatment, but crypt depth was increased (*p* < 0.001) in the duodenum of birds fed MT in contrast to CON. Broilers fed MT + 0.2% YCWE had reduced duodenal crypt depth compared with MT and did not differ from CON. The ratio of villi height to crypt depth was reduced (*p* = 0.017) in birds fed MT compared with CON while it was increased in MT + 0.2% YCWE compared with MT (*p* < 0.001) and CON (*p* = 0.006). Goblet cell count continued to be reduced (*p* = 0.015) on day 42 in the duodenum of birds fed MT compared to CON, while broilers fed MT + 0.2% had a greater (*p* = 0.019) goblet cell count than MT.

#### 2.2.4. Production Efficiency

Upon calculation of the European Production Efficiency Factor (EPEF), it was observed that broilers consuming mycotoxins in diet MT had a lower EPEF value (*p* < 0.001) than CON on day 42 (Table 4). Broilers fed MT + 0.2% YCWE had an increased EPEF (*p* = 0.003) in contrast to MT. Additionally, these birds fed YCWE during the mycotoxin challenge had a 307.9 EPEF value that was not different to birds fed CON (*p* = 0.289). 

### 2.3. Statistical Analysis 2

#### 2.3.1. Performance

The body weight of broilers fed mycotoxins and five different titers of YCWE were not impacted by treatment (Table 5). Although the main treatment effect was not significant, by day 35 a quadratic response (*p* < 0.025) could be observed. Feed intake was also not impacted by treatment for the first 14 days (Table 5). On day 21, there was a cubic response of YCWE inclusion with broilers consuming 0.25%, 0.05% and 0.10% having greater (*p* = 0.000) cumulative feed intake. A cubic response was also observed on day 28, although only birds consuming YCWE at 0.10% and 0.40% had increased feed intake. The cubic response continued through days 35 and 42, with treatments containing 0.025%, 0.05%, 0.10% and 0.40% YCWE along with the mycotoxin challenge having greater (*p* = 0.000) feed intake. The FCR was not impacted during the first 35 days (Table 5). However, on day 42, there was a quadratic response for YCWE inclusion during mycotoxin challenge, with MT + 0.20% YCWE having a lower FCR than MT and MT + 0.025% YCWE.

#### 2.3.2. Blood Parameters

There was no impact of YCWE on blood parameters at 21 (Table 6) or 42 days (Table 7). However, on day 42 there was a tendency (*p* = 0.061) for decreasing LCAT with increasing YCWE inclusion.

#### 2.3.3. Intestinal Morphology

**Figure 3:**
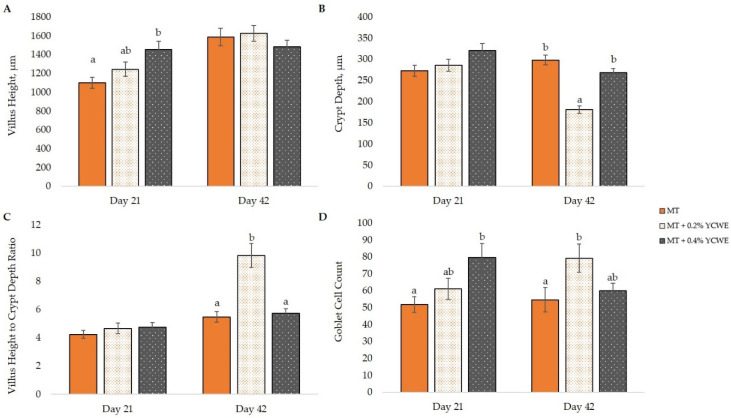
Effect of a natural multi-mycotoxin challenge and a yeast cell wall extract (YCWE) on duodenal morphology of villus height (**A**), crypt depth (**B**), villus height to crypt depth ratio (**C**) and goblet cell count (**D**) of broilers (mean ± SE). MT: diet containing a mycotoxin challenge; MT + 0.2% YCWE: MT + 0.2% YCWE; MT + 0.4% YCWE. Different letters above bars indicate significant difference between treatments (*p* < 0.05).

There was a liner response of inclusion of YCWE for duodenal villi height on day 21, with broilers fed MT + 0.4% YCWE having greater (*p* = 0.001) villi height than MT (Figure 3). This response was also observed for goblet cell count, with an increased (*p* = 0.004) number of cells in the duodenum of birds fed MT + 0.4% YCWE than MT. On day 42, there was no treatment effect for duodenum villi height (Figure 3), but broilers fed MT + 0.2% YCWE had reduced duodenal crypt depth (*p* < 0.001), increased ratio of villi height to crypt depth (*p* = 0.001) and greater goblet cell count (*p* = 0.011).

#### 2.3.4. Production Efficiency

Inclusion of 0.025% to 0.4% YCWE during the mycotoxin challenge did not have a significant main effect impact on EPEF, although a quadratic polynomial contrast response was observed (*p*= 0.009) due to the presence of the MT + 0.4% YCWE treatment (Table 8).

## 3. Discussion

Mycotoxins are a cause for concern for the poultry industry as they may reduce poultry performance and increase susceptibility to diseases [12]. Although there are many research publications investigating the effect of high concentrations of mycotoxins on poultry, fewer trials look at chronic low-level mycotoxin consumption, particularly in a setting close to commercial production conditions, to understand how broiler performance and health may be impacted. A recent publication has indicated that broilers may be sensitive to the presence of moderate DON levels (2.27 to 5.84 mg/kg) with negative effects on gain and feed efficiency [13]. This research, however, was not conducted under commercial conditions and birds were only fed for 7 days. In our current research, it was observed that mycotoxin consumption had some impact on the performance of young chicks in the first 7 days but there was a significant effect on performance and health during the grower and finisher phases following chronic dietary consumption.

Although individual mycotoxins can certainly play a role in performance, the presence of multiple mycotoxins in feeds may potentiate their negative impact on the birds due to interactions between mycotoxins [14]. The FDA and the EFSA provide regulatory guidance for mycotoxins such as AFB1, DON and fumonisin [5]. Concentrations in feed of these three mycotoxins in our trial were below the maximum levels recommended by the FDA and EFSA for complete poultry feed. However, these maximum recommendations are for individual mycotoxins and do not take into account their presence in a mixture. Analysis of feed samples by ultra-performance liquid chromatography coupled to tandem mass spectrometry (UPLC-MS/MS) in our research showed that mycotoxin contaminated diets contained on average 6.2 different types of mycotoxins with the majority of these being *Fusarium* mycotoxins. This co-occurrence of mycotoxins potentially increases the potential of seeing adverse effects in birds fed with those diets, even when individual mycotoxin concentrations were below regulatory limits or recommendations.

The mycotoxin analysis of dietary samples did show some variation between treatments regarding mycotoxin levels. Although mycotoxin content did vary, the trends remained consistent. Mycotoxins are expected to fluctuate naturally throughout feedstuffs and feeds, with some portions containing high levels of mycotoxins and others containing little or no mycotoxin contamination [15]. As a result, some variation in mycotoxin concentrations between samples may be expected. To address this natural variation, a comprehensive mixing process and sampling protocol was implemented throughout each batch.

To address the impacts of mycotoxins and YCWE on broilers, two different methods of post-experimental statistical analysis were conducted. In the first, the efficacy of the YCWE mitigation in the condition of a mycotoxin challenge was assessed. The 0.2% inclusion level was selected as a point of comparison as this level of inclusion represents the recommended rate of use for the given challenge as it relates to the mycotoxin contamination detected in the feed. Additionally, this inclusion rate has been used by many authors in the specific field. Thus, 0.2% YCWE represents a key vantage point for efficacy evaluation. Finally, the additional inclusion rates of YCWE were included in the second statistical evaluation. The second method assessed a dose response of the YCWE that reflects the evaluation of product response kinetics.

During the starter phase, the feeding of the mycotoxin contaminated ration did not impact weight gain, but feed intake was significantly lower during this period. Interestingly, FCR during the first 7 days was reduced in birds consuming MT compared with birds consuming CON. Results obtained by other researchers have shown varied effects of mycotoxins during the starter phase. Several trials found that mycotoxins such as ochratoxin A, DON, fusaric acid or zearalenone may not impact the performance of broilers during the starter period [10,16]. However, under different circumstances, mycotoxins may influence performance of young birds. For example, the weight gain of broiler chicks consuming a range of 1.7 to 12.2 mg/kg DON [17] and turkey poults consuming 1.7 mg/kg DON [18] was reduced over 21 days. Additionally, mixtures of mycotoxins at levels found in commercial productions have also been shown to suppress gain and increase FCR of starter broilers [6].

The current research was completed over a 42-day trial period to investigate the effects of chronic mycotoxin consumption by broilers. At the end of both the grower phase (day 35) and finisher phases (day 42), broilers consuming mycotoxins in MT had reduced body weight and increased FCR. These results are supported by Wang and Hogan [19], who demonstrated that broilers provided moderate DON contaminated diets later in the growth stage (22 to 34 days) had lower weight gain and increased FCR which was not observed in birds fed contaminated diets during the earlier growth stage. The results of that trial suggested broilers may be more sensitive to diets containing DON during later growth stages due to an increase in feed intake and age-related differences in update, metabolism and excretion of mycotoxins. Our research also shows a potential for age related differences in performance when broilers consume naturally contaminated feedstuffs with multiple mycotoxins (DON at 2.26 mg/kg in MT). In this case, the chronic intake of mycotoxins throughout the bird’s life may be a cause for the increase in effect as the bird ages.

While performance was altered by consumption of lower levels of mycotoxins, the measured blood parameters were minimally impacted by the presence of mycotoxins or YCWE except for ADA on day 21 which was increased in broilers fed MT. An increase in the enzyme ADA can indicate the presence of tissue damage and can play a role in inflammation due to its potential effect in decreasing adenosine, an anti-inflammatory molecule [20]. Aflatoxin consumption was shown by da Silva et al. [20] to increase ADA in quails, while Lautert et al. [21] showed that other mycotoxins may reduce ADA activity in chickens. In our research, it was observed that the consumption of mycotoxins could have played a role in increasing ADA, and subsequently causing tissue damage, although this effect was only detected on day 21 and not at the end of the trial on day 42. Regarding other blood parameters; it is previously documented by several sources that the impact of mycotoxins can be variable. For instance, the feeding of AFB1 to broilers for 21 days has been shown to reduce blood levels of globulin, albumin and total protein while increasing aspartate aminotransferase (AST) activity [22]. In contrast, other research showed that a challenge by AFB1, ochratoxin, zearalenone and T-2 toxin reduced AST at 21 days but failed to characterize any significant impact when observed at 35 days [4]. Furthermore, a diet containing only the *Fusarium* mycotoxin DON at 3 mg/kg fed over 42 days did not impact measured blood metabolites [23]. Taking these trials into account, along with our current research, it may be concluded that AFB1 has a stronger influence on blood metabolites than *Fusarium* type mycotoxins, especially as AFB1 tends to target liver metabolism more strongly. This AFB1 accumulation is shown by Moran et al. [24] who demonstrated direct hepatic accumulation of AFB1 in liver as well as metabolites that could have an impact on blood parameters.

Although the influence of mycotoxins on broiler performance increased over time, it appears that gut health may be impacted throughout the starter and finisher phases. In our trial, the consumption of mycotoxins reduced duodenal villi height at day 21 but increased duodenal crypt depth by day 42. These changes in intestinal structure have been associated with poor gut health due to potential decrease in nutrient digestibility, inflammation and higher tissue turnover [25]. Furthermore, goblet cell count was reduced in birds consuming mycotoxins on both 21 and 42 days. Goblet cells are located along the epithelial layer of villi and produce mucin that makes up the intestinal mucus layer [25]. This layer protects the intestine from damage by a variety of sources including bacterial and environmental toxins.

As a result of the mycotoxin challenge, young broilers may be more exposed to intestinal microbial infections and poor nutrient utilization which in turn may alter long term health and performance. Research with turkey poults showed that *Fusarium* mycotoxins can reduce duodenum villi height after 21 days [26]. Although villi height or crypt depth was not impacted by 42 days, villus width was reduced following mycotoxin consumption indicating that some changes in gut health may occur over time. Overall, the analysis of duodenum samples showed an impact of mycotoxin intake on the villi structure and goblet cell count, providing evidence that the chronic consumption of lower levels of multiple mycotoxins can play a role in gut health which may in turn be a factor in the observed long-term performance loss or a modification of the optimal growth rate. Furthermore, even when performance of birds consuming mycotoxins was minimally altered during the starter phase, our data show that the intestine may still be damaged.

The EPEF is a standard calculation used in many countries as a measure for determining broiler performance efficiency [27]. This calculation considers broiler body weight, FCR and livability. Higher EPEF values indicate that body weight gain is more uniform, and birds are in better health [28]. Considering the impact of mycotoxins on performance of broilers in our trial, it is logical that broilers consuming MT had a significantly lower EPEF value. Few studies have investigated the impact of mycotoxins on broiler EPEF, although one publication showed that as aflatoxin in naturally contaminated feeds increased from 280 to 1087 ppb, there was a gradual decline in EPEF [29].

Several strategies can be implemented to reduce the impact of mycotoxins on animals including agronomic techniques in the field, proper storage of feedstuffs/feeds and inclusion of feed additives that interact with mycotoxins and therefore reduce the exposure to the animal [30]. To counteract the effect of mycotoxins on the growth performance of broilers in this trial, the YCWE was included in the ration. At a feeding rate of 0.2%, broilers fed YCWE during the mycotoxin challenge had increased body weight, lower FCR and improved gut health by day 42. Furthermore, the addition of 0.2% YCWE during the mycotoxin challenge resulted in lowering of FCR to a value that was statistically the same as the CON fed birds. Furthermore, the inclusion of 0.2% YCWE during the mycotoxin challenge did lower ADA on day 21 compared with MT, to result in a similar value observed for CON fed birds. The presence of 0.2% YCWE also reduced duodenal crypt depth, increased the villi height to crypt depth ratio, and increased goblet cell count on day 42. As a result, it may be observed that 0.2% YCWE provided some protective effect against the impact of mycotoxins on tissue damage.

Calculation of EPEF for mycotoxin mitigation strategies such as YCWE could be helpful for determining product efficiency and financial benefit during a mycotoxin challenge. Although mycotoxins reduced EPEF, the birds fed MT + 0.2% YCWE in our study had a significantly improved EPEF value on day 42, indicating that the presence of the YCWE improved performance and health of birds. Furthermore, the EPEF of these broilers was not different from that of birds fed the control diet without a mycotoxin challenge. As a result, the use of YCWE at 0.2% during the mycotoxin challenge was of value. Few trials have investigated the impact of additives on EPEF during a multi-*Fusarium* mycotoxin challenge, however data from Saki et al., [31] showed that the inclusion of 0.25% of Mycosorb during an aflatoxin challenge of 1 mg/kg significantly improved a similar calculation of performance known as the European Broiler Index.

Most research with mycotoxin binding technologies investigate the ability of the strategy to reduce mycotoxin effects on the animal at one inclusion rate. Although our research did focus on one inclusion rate (0.2% YCWE) as a point of reference, it also investigated the efficiency of five different titers of YCWE during mycotoxin challenge in hopes that results may provide better assessment of the differences between YCWE inclusion rates during a challenge involving a mixture of multiple mycotoxins. Our data indicate that broilers fed different inclusion rates of 0.025% to 0.4% YCWE during the mycotoxin challenge had numerically greater but not statistically increased body weights from those fed MT by the end of the grower and finisher phases. In contrast to our observations, a different trial feeding low concentrations of multiple mycotoxins showed that inclusion of as low as 0.05% esterified glucomannan additive did statistically improve broiler performance [6].

Despite body weight was minimally altered by the titers of YCWE, inclusion of this product did alter cumulative feed intake between days 21 and 42. Generally, it was observed that broilers fed 0.025% to 0.1% YCWE had the greatest feed intake while those fed 0.2% YCWE had the lowest feed intake of all additive treatments. The latter was most similar to those birds fed only mycotoxins in MT. However, although having similar feed intake, by day 42 the broilers fed MT + 0.2% YCWE had the lowest FCR which was statistically lower than birds fed mycotoxins alone. Conflicting results have been observed for additive effects during the growing and finishing phases. In one example, broilers consuming lower concentrations of multiple mycotoxins during days 28 to 42 did not have improved FCR when given 0.05% or 0.1% esterified glucomannan, 0.5% hydrated sodium calcium aluminosilicate or 1.0% activated charcoal [6]. However, broilers consuming 0.05% esterified glucomannan had lower FCR after 35 days of consuming a diet naturally contaminated with lower to moderate levels of mycotoxins consisting primarily of AFB1 [4]. As a result of these varied responses, it may be concluded that changes to FCR may be impacted by the mycotoxin type and concentration as well as the product type and inclusion rate.

The difference in broiler performance response when given the varying titers of YCWE were expected, i.e., as YCWE inclusion increased so did the response during the mycotoxin challenge, until the inclusion rate of 0.4%. This higher inclusion rate resulted in unexpected broiler responses and quadratic or cubic statistical contrasts. To further understand the meaning of the 0.4% inclusion, we analyzed the data without this treatment (statistical analysis not shown) and observed that all other YCWE treatments had a more linear relationship. As a result, it is concluded that we cannot fully explain the response of the 0.4% YCWE treatment as it acts as an outlier but suggestions for this observation include that (1) the feed may not have been prepared as expected or (2) that at this mycotoxin risk level the inclusion of 0.4% resulted in birds that had surpassed optimal performance. Although performance was not benefited with 0.4% YCWE, we did not observe any negative effects of this inclusion on blood parameters of gut health metrics. Furthermore, previous research shows no negative effects of YCWE up to 0.8% on body weight, FCR or mortality of broilers [10].

To counteract the effect of mycotoxins on the gastrointestinal tract, the addition of adsorbent materials such as YCWE to the ration may be beneficial due to their primary interaction with mycotoxins occurring in the intestinal tract. When broilers consumed 0.2% or 0.4% YCWE during the mycotoxin challenge, the impacts of mycotoxins on the duodenum were reduced. Interestingly, on day 21 at the end of the starter phase, an inclusion of 0.4% had a more significant impact on improving duodenum structure during the mycotoxin challenge than 0.2%, although numerical improvement was still observed with a 0.2% YCWE inclusion. These results may indicate that a higher product inclusion rate may have been necessary to significantly reduce negative effects of mycotoxins on the intestine during the starter phase. This occurrence is perhaps due to the high rate of development of this organ system in young birds [32]. A similar effect of product inclusion was observed by Vartiainen et al. [10], where broilers fed ochratoxin A (OTA) at 0.1 mg/kg feed had increased OTA deposition in the liver that was numerically lowered with inclusion of 2.0 kg/ton of the YCWE, and significantly reduced with inclusion of 4.0 kg/ton YCWE to the ration. Conversely, the inclusion of 0.2% glucomannan polymer to a turkey diet containing *Fusarium* mycotoxin over 21 days was enough to significantly improve duodenum villus height [23]. The researchers in the latter trial did not investigate an inclusion of 0.4%, so the difference between product inclusion cannot be determined in that case.

Intestinal damage from mycotoxins continued during the growing and finishing phases in our trial. By day 42, the inclusion of 0.2% YCWE resulted in significant improvement in villi structure and goblet cell count with no additional effect of 0.4% YCWE inclusion. Interestingly, at this time point the broilers consuming MT + 0.2% YCWE also had the greatest body weight and lowest FCR of all birds consuming mycotoxin contaminated diets. As a result, it may be concluded that the intestinal structure of young birds was impacted to a greater extent and needed additional product supplementation rate, while the risk to broilers continues as they age but a YCWE inclusion rate of 0.2% may be sufficient to mitigate the experimental challenge investigated in our study.

## 4. Conclusions

Mixtures of mycotoxins are common in naturally contaminated feedstuffs, and these mycotoxins may play a role in broiler performance and health even at lower dietary concentrations. Given the increase in significant effects of mycotoxins on broiler performance during the grower and finisher phases in our trial, it is suggested that the co-contamination of mycotoxins at levels below FDA or EFSA guidelines may result in significant effects on broiler performance and health when consumed chronically over time. To counteract a multi-mycotoxin challenge, YCWE may play a valuable role. Considering a range of 0.025% to 0.4% YCWE, it was observed that all inclusion rates tested provided some benefit to birds but an inclusion rate of 0.2% YCWE provided the most statistically significant results and the overall best response at improving performance and gut health in broilers exposed to the investigated mycotoxin challenge. As a result, it is concluded that the use of YCWE could be a useful approach to mitigate the negative effects of mycotoxins in poultry.

## 5. Materials and Methods

### 5.1. Experimental Design

One thousand six hundred one-day-old male Cobb X Cobb chicks (Cobb-Vantress hatchery, Cleveland, GA, USA) were randomly allocated to 64 pens of 25 chickens. Birds were housed from day 0 to 42 at Southern Poultry Research, Inc. (Athens, GA, USA). Treatments were replicated in 8 blocks with 8 treatments within each block following a randomization procedure for pen assignment. Chicks were spray vaccinated on day of hatch with Cocci-Vac “B” at the manufacturer recommended dose. All commercially applicable vaccinations were administered at the hatchery.

The test house is divided into pens of equal size, arranged along a central aisle. The pen was the experimental unit. Each pen was 1.88 m^2^ (1.37 × 1.37 m) with 0.61 m high side walls and a bottom 15 cm foot being of solid wood to prevent bird migration. All flooring of each pen had approximately 10 cm of clean pine shavings. Environmental conditions were monitored during the trial to provide optimum temperature for the age of the birds with 23 h of light and 1 h of dark per day. Standard floor pen management practices were used throughout the experiment. Animals and housing facilities were inspected twice daily, observing and recording general health status, feed and water supply and temperature. Any dead birds were removed and pen number, date of mortality, weight, and diagnosis were recorded. Animal care and use protocols were approved by the Southern Poultry Research Institutional Animal Care and Use Committee (Southern Poultry Research Inc., Athens, GA, USA).

Feed and water were provided ad libitum throughout the trial. Feed was weighed by pen at weekly intervals and bird weights by pen were recorded on days 0, 7, 14, 21, 28, 35, and 42. Means for body weight, feed consumption and feed conversion ratio were calculated.

The experimental animal protocol has been reviewed and approved by IACUC on December 14, 2016 for the study Number: 16-E-10820.

### 5.2. Experimental Diets

Diets were based on current commercial standards and were formulated to be isocaloric and isonitrogenous (Table 9). All feeds were mash and followed a 3-phase feeding system including the starter from day of hatch until day 21, grower from day 21 to 35, and finisher from day 35 through the end of the trail at day 42. Broilers were fed experimental diets based on their assigned treatment group: (1) control with minimal mycotoxins (CON); (2) diet containing mycotoxin challenge from mycotoxin contaminated DDGS (MT); (3) CON + 0.2% YCWE (Mycosorb A+, Alltech, Inc., Nicholasville, KY, USA); (4) MT + 0.025% YCWE; (5) MT + 0.05% YCWE; 6) MT + 0.1% YCWE; (7) MT + 0.2% YCWE; and (8) MT + 0.4% YCWE.

In order to ensure similar nutrient and mycotoxin levels between dietary treatments while limiting cross contamination of the mycotoxins, two mixers were used. The large 1.5-t mixer allowed for large volume mixing, while a second smaller mixer allowed for complete clean out and removal of all feed. These systems were flushed with soybean meal between mixes. In addition, two basal diets per feeding phase were formulated, one which would be the source for control feed and the other for mycotoxin contaminated feed. Feed ingredients for control and contaminated diets were from the same source except for distillers dried grains with solubles (DDGS) which was included to achieve target mycotoxin concentrations in the final rations. Highly contaminated DDGS was included in the mycotoxin treatments, while the lower risk control diet contained a different DDGS source with minimal mycotoxin contamination. Initial mixing was completed for the two basal diets prior to DDGS addition. Following mixing, the first basal diet was split into two batches (control without and with YCWE) which were added to the smaller mixer along with the appropriate amount of low mycotoxin DDGS. This process was repeated for the second basal mix, which was split into six batches (mycotoxin without or with varying inclusion levels of YCWE) with additional higher risk DDGS added. Diets for all three phases followed the same procedure described above. For each diet, three subsamples were collected during feed production (beginning, middle, and end) and were mixed to form a composite sample.

All treatments from the starter phase, including the basal ration used to form other diets, were analyzed for the presence of 38 mycotoxins by UPLC-MS/MS (Waters Acquity TQD, Waters Corp., Milford, MA, USA) at the Alltech 37+^TM^ Analytical Laboratory (ISO/IEC 17025:2005 official accreditation (No. 79481), Nicholasville, KY, USA) following methods described by Jackson et al. [33]. Samples of CON and MT diets from the grower and finisher phases were also analyzed for mycotoxins. Analysis of each sample was completed in triplicate, with final mycotoxin averages reported.

### 5.3. Serum Collection

On day 21 and 42, blood samples were collected from 3 birds (1 bird/pen) from each of the 8 treatments. Birds were bled by cardiac puncture, and blood samples were collected in vacutainer serum blood collection tubes, left at room temperature for a minimum of two hours to coagulate before centrifuging. Serum were then collected and flash frozen in liquid nitrogen and stored at −80°C. Concentrations of adenosine deaminase (Diazyme, Diazyme, Poway, CA, USA), aspartate transaminase (Empire Genomics, Idlabs Biotechnology, Buffalo, NY, USA), alanine transaminase (Empire Genomics, Idlabs Biotechnology, Buffalo, NY, USA), apolipoprotein B (Elabscience, Elabscience, Houston, TX, USA), lecithin-cholesterol acyltransferase (NeoScientific, Abclonal, Woburn, MA, USA), total cholesterol (Enzychrom, antibody-antibodies.com, Kampenhout, Belgium), phospholipid transfer protein (Elabscience, antibody-antibodies.com, Kampenhout, Belgium), free cholesterol (BlueGene, Antibodies-online, Limerick, PA, USA) and glucose-6 phosphate dehydrogenase (Abcam, Abcam, Cambridge, MA, USA) were analyzed. Analysis of blood samples was completed at the Alltech Center for Animal Nutrigenomics and Applied Animal Nutrition (Nicholasville, KY, USA).

### 5.4. Tissue Collection

On day 21 and 42, 3 birds (1 bird/pen) from treatments CON, MT, MT + 0.2% YCWE and MT + 0.4% YCWE were euthanized by cervical dislocation according to American Veterinary Medical Association guidelines [34] and tissue samples of duodenum were collected. Tissues samples were stored in 10% formalin and processed using a tissue processor (Microm STP 120, WalTham, MA, USA) by subjecting samples to several fixative solutions: 90min in five incremental ethanol titers (70% to 100%) before being placed in Citra Clear™ (Stat lab, McKinney, TX, USA) for 3hrs followed by paraffin waxing for another 3-h cycle. Tissue samples were then orientated, embedded (Microm EC- 350-2, WalTham, MA, USA) and trimmed (HM 340 E Microtome, WalTham, MA, USA) before staining. Sample slices were placed in a 45 °C water bath before mounting into microscope slide (Mercedes Medical, Lakewood Ranch, FL, USA). Specimens were oven-heated at 60 °C for 2 min before staining. Alcian Blue and Periodic Acid-Schiff reaction were used for staining according to [35]. Briefly, after deparaffinization using Citra Clear™, ethanol, and rehydration with distilled water, slides were then saturated with Alcian blue (Sigma, St. Louis, MO, USA), rinsed and saturated with periodic acid (Sigma, St. Louis, MO, USA) for 10 min, rinsed and saturated with Schiff’s reagent (Sigma, St. Louis, MO, USA) for 15 min. Dehydration was then performed using again several ethanol titers, washing steps and in Citra Clear™ for 5 min. Specimen were dried over 24hr at room temperature. Histological samples were observed under a light microscope (Nikon Eclipse E400, Nikon Corporation Instruments Company, Tokyo, Japan). For every sample, ten complete villi sections were captured and villus length, crypt depth were measured, villus-to-crypt ratio was calculated using Image J software (NIH, Bethesda, MD, USA). Goblet cell count was also performed on the same samples and for 10 complete villi per sample. Analysis of intestinal samples was completed at the Alltech Center for Animal Nutrigenomics and Applied Animal Nutrition (Nicholasville, KY, USA).

### 5.5. Efficiency Calculation

At the completion of the study, the European Production Efficiency Factor (EPEF) was calculated to assess the performance and economic efficiency of broilers fed different experimental rations. The EPEF was calculated using the following formula suggested by Marcu et al. [27],

(1) $$\mathrm{EPEF}\;=\;\frac{\mathrm{viability}\times \mathrm{BW}}{\mathrm{Age}\times \mathrm{FCR}}\;\times \;100$$

where the viability is expressed in %; BW is the animal body weight in kg; the age in days; and FCR is the feed conversion ratio expressed in kg feed consumed by kg of body weight gain.

### 5.6. Statistical Analysis

#### 5.6.1. Statistical Analysis 1

For both post-experiment processing approaches, data from each pen were the experimental unit. Data for statistical analysis 1 were analyzed according to a one-way factorial design of analysis of variance (ANOVA) procedure (SPSS Statistics software, IBM Corp, Version 21.0, Armonk, NY, USA, 2018) according to the model: yij = µ + ai + eij; where yij = jth observation subjected to treatment i; µ = overall mean; ai = effect of treatment; eij = residual error. Before evaluation, the data were tested for homogeneity of variance (Levene’s test) and normality (Shapiro’s test). Hypothesis-driven planned contrast comparisons (Bonferroni-type based on Student’s *t*-distribution) were performed for specific treatment group means using differences of least squares means at the *p* < 0.05 level if a significant *F*-test statistic was obtained from an ANOVA. The data are presented as the mean ± standard error of the mean (SEM) and *F*-test statistical results were considered significant at *p* < 0.05.

#### 5.6.2. Statistical Analysis 2

For statistical analysis 2, the effect of titration of YCWE (0 to 0.4%) during the mycotoxin challenge was investigated. The ANOVA polynomial contrasts (linear, quadratic, and cubic) were used to analyze the trend in the dependent variable across the ordered levels of the YCWE in the diet. Statistical analyses for best fit on the response involved simple *t*-tests where the trend relation was declared significant for *p*-values lower than 0.05 (in two-tailed tests). The data are presented as the mean ± standard error of the mean (SEM).

## Figures and Tables

**Table 1:** Analyzed mycotoxin content (µg/kg) of experimental diets.

Treatment ^2^	Mycotoxin Content, µg/kg ^1^								
	AFB1	DON	3-DON	15-DON	HT2	FA	FB1	FB2	FB3
**Starter Ration**									
Basal		187.0			80.2	170.3			
CON	− ^3^	119.8	−	−	−	59.0	1128.2	−	−
MT	−	2263.8	26.1	41.7	−	59.4	−	−	−
CON + 0.2% YCWE	1.3	695.4	−	−	165.2	−	1037.41	−	−
MT + 0.025% YCWE	−	1264.0	−	−	115.2	31.4	770.3	261.5	231.6
MT + 0.05% YCWE	−	1665.2	18.5	25.3	−	51.0	1136.0	4101.2	420.2
MT + 0.1% YCWE	−	1684.4	18.1	−	−	67.1	2146.2	629.6	558.9
MT + 0.2% YCWE	−	1456.9	17.1	−	95.0	45.5	1226.4	874.4	512.6
MT + 0.4% YCWE	−	1937.2	22.0	30.1	−	55.7	1138.7	2122.5	1422.5
**Grower Ration**									
CON	−	659.3	−	−	−	201.3	745.3	172.8	40.1
MT	−	2134.7	38.5	−	−	103.5	−	−	−
**Finisher Ration**									
CON	−	1111.5	−	−	55.9	169.9	−	−	−
MT	−	2750.8	46.2	56.0	−	116.0	−	47.9	−

^1^ AFB1: aflatoxin B1; DON: deoxynivalenol; 3-DON: 3-acetyl-deoxynivalenol; 15-DON: 15-acetyl-deoxynivalenol; HT2: HT-2 toxin; FA: fusaric acid; FB1: fumonisin B1; FB2: fumonisin B2; FB3: fumonisin B3. Levels of mycotoxins are reported only for those above the limit of quantitation of each mycotoxin; ^2^ CON: control diet with minimal mycotoxins; MT: diet containing mycotoxin distillers dried grains with solubles (DDGS); CON + 0.2% YCWE: CON + 0.2% yeast cell wall extract (YCWE, Mycosorb A+^®^, Alltech, Inc., Nicholasville, KY, USA); MT + 0.025% YCWE; MT + 0.05% YCWE; MT + 0.1% YCWE; MT + 0.2% YCWE; and MT + 0.4% YCWE; ^3^ −: not detectable.

**Table 2:** Effect of a natural multi-mycotoxin challenge on performance of broilers fed diet with or without addition of a yeast cell wall extract (YCWE) (mean ± standard error (SE)).

Treatments ^1^	Trial Day					
	7	14	21	28	35	42
**Body Weight, kg**						
CON	0.119 ± 0.003	0.334 ± 0.015	0.703 ± 0.015	1.204 ± 0.032	1.801 ± 0.034	2.333 ± 0.043
MT	0.123 ± 0.001	0.347 ± 0.004	0.691 ± 0.009	1.184 ± 0.010	1.651 ± 0.014	2.124 ± 0.032
CON + 0.2% YCWE	0.119 ± 0.002	0.344 ± 0.006	0.707 ± 0.013	1.217 ± 0.017	1.812 ± 0.020	2.388 ± 0.028
MT + 0.2% YCWE	0.120 ± 0.002	0.341 ± 0.009	0.695 ± 0.010	1.173 ± 0.014	1.707 ± 0.02	2.245 ± 0.032
	Treatment *p*-Values					
Main effect	0.636	0.817	0.802	0.453	<0.000	<0.000
	Contrast *p*-Values					
CON vs. MT	0.289	0.359	0.520	0.507	0.000	0.000
CON vs. CON + 0.2% YCWE	0.920	0.509	0.806	0.649	0.729	0.269
CON vs. MT + 0.2% YCWE	0.815	0.644	0.669	0.308	0.009	0.082
MT vs. MT + 0.2% YCWE	0.406	0.644	0.828	0.717	0.110	0.020
CON + 0.2% YCWE vs. MT + 0.2% YCWE	0.738	0.841	0.502	0.145	0.004	0.007
**Feed Intake, kg**						
CON	3.087 ± 0.212	11.268 ± 0.174	22.456 ± 0.197	39.806 ± 0.525	62.225 ± 0.959	83.45 ± 1.406
MT	2.487 ± 0.081	10.612 ± 0.178	21.475 ± 0.194	38.25 ± 0.761	59.200 ± 1.154	78.712 ± 1.756
CON + 0.2% YCWE	2.593 ± 0.175	11.456 ± 0.314	23.493 ± 0.339	43.175 ± 0.628	67.468 ± 1.023	92.568 ± 1.525
MT + 0.2% YCWE	2.45 ± 0.124	10.781 ± 0.295	21.643 ± 0.42	37.393 ± 0.764	60.862 ± 0.85	82.137 ± 1.202
	Treatment *p*-Values					
Main effect	0.027	0.076	<0.001	<0.001	<0.001	<0.001
	Contrast *p*-Values					
CON vs. MT	0.011	0.073	0.030	0.115	0.042	0.032
CON vs. CON + 0.2% YCWE	0.034	0.599	0.022	0.002	0.001	0.000
CON vs. MT + 0.2% YCWE	0.008	0.178	0.069	0.018	0.345	0.537
MT vs. MT + 0.2% YCWE	0.867	0.636	0.697	0.379	0.251	0.114
CON + 0.2% YCWE vs. MT + 0.2% YCWE	0.522	0.066	0.000	0.002	0.000	0.000
**Feed Conversion Ratio**						
CON	0.894 ± 0.051	1.357 ± 0.054	1.365 ± 0.024	1.486 ± 0.022	1.593 ± 0.012	1.676 ± 0.007
MT	0.702 ± 0.018	1.245 ± 0.026	1.356 ± 0.026	1.469 ± 0.037	1.675 ± 0.024	1.757 ± 0.011
CON + 0.2% YCWE	0.756 ± 0.051	1.29 ± 0.030	1.343 ± 0.019	1.479 ± 0.018	1.568 ± 0.01	1.665 ± 0.007
MT + 0.2% YCWE	0.71 ± 0.041	1.262 ± 0.031	1.332 ± 0.025	1.418 ± 0.028	1.622 ± 0.012	1.696 ± 0.011
	Treatment *p*-Values					
Main effect	0.013	0.178	0.775	0.312	<0.001	<0.001
	Contrast *p*-Values					
CON vs. MT	0.004	0.042	0.782	0.678	0.001	0.000
CON vs. CON + 0.2% YCWE	0.030	0.213	0.509	0.856	0.278	0.454
CON vs. MT + 0.2% YCWE	0.005	0.081	0.336	0.093	0.210	0.140
MT vs. MT + 0.2% YCWE	0.899	0.754	0.490	0.197	0.028	0.000
CON + 0.2% YCWE vs. MT + 0.2% YCWE	0.451	0.595	0.760	0.130	0.024	0.031

^1^ CON: control diet with minimal mycotoxins; MT: diet containing mycotoxin DDGS; CON + 0.2% YCWE; MT + 0.2% YCWE.

**Table 3:** Effect of a natural multi-mycotoxin challenge on blood parameters of broilers fed diet with or without addition of a yeast cell wall extract (YCWE) (mean ± SE).

Treatments ^1^	Item ^2^								
	ADA (U/L)	AST (U/L)	ALT (U/L)	Apo B (ng/mL)	LCAT (ng/mL)	TCHOL (ng/mL)	PLTP (ng/mL)	FCHOL (ng/mL)	G6PDH (mU/mL)
**Day 21**									
CON	38.29 ± 3.47	155.31 ± 7.40	23.35 ± 2.76	91.24 ± 35.01	0.72 ± 0.01	11.99 ± 0.41	11.87 ± 3.60	136.31 ± 11.86	30.42 ± 0.71
MT	55.39 ± 4.12	159.01 ± 15.09	19.86 ± 4.49	83.02 ± 9.58	0.53 ± 0.02	11.24 ± 0.51	13.38 ± 2.42	105.18 ± 16.54	28.81 ± 2.74
CON + 0.2% YCWE	58.02 ± 4.00	167.53 ± 8.29	27.87 ± 11.22	84.57 ± 7.03	0.64 ± 0.03	14.25 ± 0.71	13.49 ± 0.80	83.38 ± 7.46	30.62 ± 0.57
MT + 0.2% YCWE	42.75 ± 1.44	180.74 ± 24.08	23.8 ± 4.80	90.91 ± 9.46	0.67 ± 0.06	12.91 ± 1.12	15.28 ± 3.14	53.13 ± 12.62	33.9 ± 0.73
	Treatment *p*-Values								
Main effect	0.009	0.664	0.864	0.984	0.052	0.093	0.849	0.009	0.186
	Contrast *p*-Values								
CON vs. MT	0.008	0.868	0.720	0.768	0.010	0.497	0.704	0.117	0.469
CON vs. CON + 0.2% YCWE	0.004	0.587	0.644	0.811	0.211	0.064	0.684	0.18	0.926
CON vs. MT + 0.2% YCWE	0.385	0.273	0.962	0.990	0.410	0.408	0.399	0.002	0.138
MT vs. MT + 0.2% YCWE	0.032	0.344	0.686	0.777	0.038	0.152	0.633	0.019	0.042
CON + 0.2% YCWE vs. MT + 0.2% YCWE	0.014	0.557	0.677	0.820	0.637	0.239	0.652	0.127	0.159
**Day 42**									
CON	27.62 ± 6.98	222.74 ± 14.23	10.46 ± 1.94	66.72 ± 7.11	0.77 ± 0.06	11.48 ± 1.03	2.38 ± 1.29	35.11 ± 6.46	29.74 ± 1.07
MT	46.22 ± 12.54	227.06 ± 8.28	7.01 ± 2.39	95.49 ± 16.49	0.7 ± 0.04	11.44 ± 0.82	1.48 ± 0.59	No data ^3^	28.6 ± 1.23
CON + 0.2% YCWE	40.94 ± 7.55	242.74 ± 15.43	11.65 ± 2.22	73.61 ± 8.95	0.73 ± 0.09	9.74 ± 0.68	2.34 ± 0.78	91.14 ± 0.00	30.24 ± 0.70
MT + 0.2% YCWE	50.39 ± 10.47	284.96 ± 42.74	7.2 ± 3.38	101.72 ± 13.57	0.67 ± 0.01	11.05 ± 0.18	1.87 ± 0.97	No data	28.89 ± 2.71
	Treatment *p*-Values								
Main effect	0.422	0.317	0.509	0.203	0.709	0.378	0.893	−	0.881
	Contrast *p*-Values								
CON vs. MT	0.210	0.903	0.366	0.132	0.451	0.973	0.521	−	0.631
CON vs. CON + 0.2% YCWE	0.358	0.575	0.750	0.698	0.697	0.140	0.973	−	0.834
CON vs. MT + 0.2% YCWE	0.134	0.106	0.392	0.075	0.293	0.694	0.714	−	0.719
MT vs. MT + 0.2% YCWE	0.768	0.129	0.959	0.726	0.748	0.718	0.779	−	0.903
CON + 0.2% YCWE vs. MT + 0.2% YCWE	0.509	0.252	0.252	0.140	0.491	0.254	0.739	−	0.572

^1^ ADA: adenosine deaminase; AST: aspartate aminotransferase; ALT: alanine aminotransferase; Apo B: apolipoprotein B; LCAT: lecithin cholesterol acyltransferase; TCHOL: total cholesterol; PLTP: plasma phospholipid-transfer protein; FCHOL: free cholesterol; G6PDH: glucose 6-phosphate dehydrogenase; ^2^ CON: control diet without mycotoxins; MT: diet containing mycotoxin DDGS; CON + 0.2% YCWE; NC + 0.2% YCWE; ^3^ No data: readings were out of range causing data to be unavailable.

**Table 4:** Effect of a natural multi-mycotoxin challenge on the European Poultry Production Efficiency (EPEF) value for broilers fed diet with or without addition of a yeast cell wall extract (YCWE) (mean ± SE).

Treatments ^1^	EPEF Values
CON	319.03 ± 6.86
MT	273.78 ± 8.76
CON + 0.2% YCWE	332.68 ± 3.26
MT + 0.2% YCWE	307.87 ± 8.85
	Treatment *p*-Values
Main effect	<0.001
	Contrast *p*-Values
CON vs. MT	<0.001
CON vs. CON + 0.2% YCWE	0.197
CON vs. MT + 0.2% YCWE	0.289
MT vs. MT + 0.2% YCWE	0.003
CON + 0.2% YCWE vs. MT + 0.2% YCWE	0.023

^1^ CON: control diet with minimal mycotoxins; MT: diet containing mycotoxin DDGS; CON + 0.2% YCWE: CON + 0.2% YCWE; MT + 0.2% YCWE.

**Table 5:** Effect of a yeast cell wall extract (YCWE) on performance of broilers during a natural multi-mycotoxin challenge (mean ± SE).

Treatments ^1^	Trial Day					
	7	14	21	28	35	42
**Body Weight, kg**						
MT	0.123 ± 0.001	0.347 ± 0.004	0.691 ± 0.009	1.184 ± 0.010	1.651 ± 0.014	2.124 ± 0.032
MT + 0.025% YCWE	0.121 ± 0.004	0.340 ± 0.010	0.677 ± 0.017	1.163 ± 0.023	1.690 ± 0.006	2.200 ± 0.025
MT + 0.05% YCWE	0.119 ± 0.001	0.339 ± 0.004	0.685 ± 0.007	1.154 ± 0.018	1.667 ± 0.022	2.173 ± 0.037
MT + 0.1% YCWE	0.120 ± 0.003	0.350 ± 0.009	0.687 ± 0.012	1.189 ± 0.018	1.704 ± 0.036	2.214 ± 0.036
MT + 0.2% YCWE	0.120 ± 0.002	0.341 ± 0.009	0.695 ± 0.010	1.173 ± 0.014	1.707 ± 0.020	2.245 ± 0.032
MT + 0.4% YCWE	0.116 ± 0.002	0.331 ± 0.008	0.674 ± 0.007	1.146 ± 0.017	1.635 ± 0.030	2.146 ± 0.052
	Treatment *p*-Values					
Main effect	0.714	0.669	0.752	0.469	0.211	0.227
	Polynomial Contrast *p*-Values					
Linear	0.159	0.214	0.530	0.263	0.373	0.988
Quadratic	0.979	0.580	0.343	0.431	0.024	0.019
Cubic	0.419	0.975	0.399	0.626	0.923	0.930
**Feed Intake, kg**						
MT	2.487 ± 0.081	10.612 ± 0.178	21.475 ± 0.194 ^a^	38.25 ± 0.761 ^ab^	59.200 ± 1.154 ^a^	78.712 ± 1.756 ^a^
MT + 0.025% YCWE	2.7 ± 0.133	11.337 ± 0.199	23.312 ± 0.298 ^c^	40.925 ± 1.221 ^abc^	67.043 ± 1.049 ^c^	90.956 ± 1.247 ^c^
MT + 0.05% YCWE	2.525 ± 0.118	11.475 ± 0.295	23.018 ± 0.286 ^bc^	40.893 ± 0.783 ^abc^	65.293 ± 1.222 ^bc^	88.506 ± 1.614 ^bc^
MT + 0.1% YCWE	2.737 ± 0.145	11.518 ± 0.376	23.187 ± 0.366 ^c^	42.55 ± 0.808 ^c^	65.862 ± 1.328 ^c^	88.662 ± 1.723 ^bc^
MT + 0.2% YCWE	2.45 ± 0.124	10.781 ± 0.295	21.643 ± 0.42 ^ab^	37.393 ± 0.764 ^a^	60.862 ± 0.85 ^ab^	82.137 ± 1.202 ^ab^
MT + 0.4% YCWE	2.443 ± 0.129	11.006 ± 0.34	22.693 ± 0.501 ^abc^	41.056 ± 0.707 ^bc^	64.531 ± 1.139 ^bc^	87.262 ± 1.703 ^bc^
	Treatment *p*-Values					
Main effect	0.382	0.153	0.001	0.001	0.000	0.000
	Polynomial Contrast *p*-Values					
Linear	0.266	0.599	0.859	0.767	0.860	0.680
Quadratic	0.705	0.554	0.858	0.518	0.976	0.825
Cubic	0.174	0.009	0.000	0.000	0.000	0.000
**Feed Conversion Ratio**						
MT	0.702 ± 0.018	1.245 ± 0.026	1.356 ± 0.026	1.469 ± 0.037	1.675 ± 0.024	1.757 ± 0.011 ^b^
MT + 0.025% YCWE	0.778 ± 0.039	1.292 ± 0.028	1.387 ± 0.023	1.449 ± 0.022	1.661 ± 0.01	1.762 ± 0.015 ^b^
MT + 0.05% YCWE	0.73 ± 0.024	1.308 ± 0.019	1.356 ± 0.014	1.463 ± 0.018	1.641 ± 0.01	1.734 ± 0.012 ^ab^
MT + 0.1% YCWE	0.803 ± 0.056	1.288 ± 0.025	1.377 ± 0.026	1.492 ± 0.012	1.644 ± 0.003	1.728 ± 0.011 ^ab^
MT + 0.2% YCWE	0.71 ± 0.041	1.262 ± 0.031	1.332 ± 0.025	1.418 ± 0.028	1.622 ± 0.012	1.696 ± 0.011 ^a^
MT + 0.4% YCWE	0.727 ± 0.028	1.28 ± 0.019	1.348 ± 0.015	1.475 ± 0.008	1.65 ± 0.016	1.732 ± 0.021 ^ab^
	Treatment *p*-Values					
Main effect	0.348	0.567	0.578	0.348	0.203	0.034
	Polynomial Contrast *p*-Values					
Linear	0.649	0.952	0.311	0.972	0.271	0.070
Quadratic	0.615	0.837	0.648	0.311	0.023	0.005
Cubic	0.088	0.093	0.272	0.122	0.846	0.554

^1^ MT: diet containing mycotoxin DDGS; MT + 0.025% YCWE: MT + 0.025% YCWE; MT + 0.05% YCWE; MT + 0.1% YCWE; MT + 0.2% YCWE; and MT + 0.4% YCWE. Means within a column with no common letter are significantly different (*p* < 0.05). Different superscript letters indicate significant difference between treatments (*p* < 0.05).

**Table 6:** Effect of a yeast cell wall extract (YCWE) on day 21 blood parameters of broilers during a natural multi-mycotoxin challenge (mean ± SE).

Treatments ^1^	Item ^2^								
	ADA (U/L)	AST (U/L)	ALT (U/L)	Apo B (ng/mL)	LCAT (ng/mL)	Total Chol (ng/mL)	PLTP (ng/mL)	Free Chol (ng/mL)	G6PDH (mU/mL)
MT	55.39 ± 4.12	159.01 ± 15.09	19.86 ± 4.49	83.02 ± 9.58	0.53 ± 0.02	11.24 ± 0.51	13.38 ± 2.42	105.18 ± 16.54	28.81 ± 2.74
MT + 0.025% YCWE	42.75 ± 6.82	157.9 ± 26.08	18.25 ± 1.11	80.35 ± 28.29	0.62 ± 0.07	14.24 ± 1.43	16.66 ± 8.51	85.5 ± 28.11	29.21 ± 4.81
MT + 0.05% YCWE	55.8 ± 8.22	149.13 ± 7.23	17.02 ± 7.93	142.91 ± 74.66	0.57 ± 0.04	14.05 ± 0.70	20.21 ± 7.59	26.86 ± 9.30	30.93 ± 1.61
MT + 0.1% YCWE	59.97 ± 9.34	179.01 ± 11.23	29.25 ± 2.11	114.35 ± 30.67	0.58 ± 0.06	14.7 ± 1.40	25.89 ± 10.77	78.27 ± 14.49	28.66 ± 1.35
MT + 0.2% YCWE	42.75 ± 1.44	180.74 ± 24.08	23.8 ± 4.80	90.91 ± 9.46	0.67 ± 0.06	12.91 ± 1.12	15.28 ± 3.14	53.13 ± 12.62	33.9 ± 0.73
MT + 0.4% YCWE	61.91 ± 7.08	186.79 ± 4.40	33.61 ± 12.29	201.13 ± 33.74	0.62 ± 0.01	14.17 ± 1.18	31.8 ± 5.72	87.42 ± 25.16	31.05 ± 1.06
	Treatment *p*-Values								
Main effect	0.245	0.550	0.461	0.256	0.582	0.321	0.451	0.122	0.667
	Polynomial Contrast *p*-Values								
Linear	0.388	0.122	0.089	0.055	0.271	0.475	0.126	0.934	0.377
Quadratic	0.327	0.551	0.923	0.446	0.301	0.551	0.766	0.085	0.359
Cubic	0.184	0.842	0.694	0.237	0.692	0.051	0.177	0.350	0.469

^1^ MT: diet containing mycotoxins; MT + 0.025% YCWE; MT + 0.05% YCWE; MT + 0.1% YCWE; MT + 0.2% YCWE; and MT + 0.4% YCWE; ^2^ ADA: adenosine deaminase; AST: aspartate aminotransferase; ALT: alanine aminotransferase; Apo B: apolipoprotein B; LCAT: lecithin cholesterol acyltransferase; Total Chol: total cholesterol; PLTP: plasma phospholipid-transfer protein; Free Chol: free cholesterol; G6PDH: glucose 6-phosphate dehydrogenase.

**Table 7:** Effect of a yeast cell wall (YCWE) on day 42 blood parameters of broilers during a natural multi-mycotoxin challenge (mean ± SE).

Treatments ^1^	Item ^2^								
	ADA (U/L)	AST (U/L)	ALT (U/L)	Apo B (ng/mL)	LCAT (ng/mL)	Total Chol (ng/mL)	PLTP (ng/mL)	Free Chol (ng/mL)	G6PDH (mU/mL)
MT	46.22 ± 12.54	227.06 ± 8.28	7.01 ± 2.39	95.49 ± 16.49	0.7 ± 0.04	11.44 ± 0.82	1.48 ± 0.59	No data ^3^	28.6 ± 1.23
MT + 0.025% YCWE	53.16 ± 6.25	248.54 ± 37.90	12.8 ± 2.00	118.5 ± 51.06	0.76 ± 0.00	9.91 ± 0.71	1.64 ± 1.46	No data	30.19 ± 0.87
MT + 0.05% YCWE	45.66 ± 5.67	239.16 ± 23.62	6.06 ± 2.36	80.5 ± 13.00	0.70 ± 0.02	10.87 ± 0.74	3.06 ± 2.88	No data	31.47 ± 0.82
MT + 0.1% YCWE	45.94 ± 3.09	212.98 ± 23.27	7.21 ± 2.22	81.27 ± 15.14	0.77 ± 0.09	9.82 ± 0.63	3.86 ± 1.67	22.8 ± 0.00	30.48 ± 0.88
MT + 0.2% YCWE	50.39 ± 10.47	284.96 ± 42.74	7.2 ± 3.38	101.72 ± 13.57	0.67 ± 0.01	11.05 ± 0.18	1.87 ± 0.97	No data	28.89 ± 2.71
MT + 0.4% YCWE	41.5 ± 11.09	206.19 ± 14.39	6.02 ± 2.05	93.72 ± 27.67	0.51 ± 0.07	11.66 ± 0.81	3.13 ± 2.14	63.85 ± 0.00	29.3 ± 1.16
	Treatment *p*-Values								
Main effect	0.951	0.433	0.417	0.913	0.061	0.344	0.906	No data	0.723
	Polynomial Contrast *p*-Values								
Linear	0.589	0.651	0.357	0.927	0.006	0.245	0.669	No data	0.633
Quadratic	0.721	0.199	0.827	0.841	0.208	0.341	0.790	No data	0.794
Cubic	0.761	0.223	0.964	0.575	0.545	0.218	0.338	No data	0.171

^1^ MT: diet containing mycotoxins; MT + 0.025% YCWE; MT + 0.05% YCWE; MT + 0.1% YCWE; MT + 0.2% YCWE; and MT + 0.4% YCWE; ^2^ ADA: adenosine deaminase; AST: aspartate aminotransferase; ALT: alanine aminotransferase; Apo B: apolipoprotein B; LCAT: lecithin cholesterol acyltransferase; Total Chol: total cholesterol; PLTP: plasma phospholipid-transfer protein; Free Chol: free cholesterol; G6PDH: glucose 6-phosphate dehydrogenase; ^3^ No data: readings were out of range causing data to be unavailable.

**Table 8:** Effect of a yeast cell wall extract (YCWE) on the European Poultry Production Efficiency (EPEF) value for broilers during a natural multi-mycotoxin challenge (mean ± SE).

Treatments ^1^	EPEF Values
MT	273.78 ± 8.76
MT + 0.025% YCWE	292.43 ± 8.57
MT + 0.05% YCWE	294.54 ± 4.88
MT + 0.1% YCWE	295.56 ± 7.19
MT + 0.2% YCWE	307.87 ± 8.85
MT + 0.4% YCWE	286.40 ± 9.94
	Treatment *p*-Values
Main effect	0.117
	Polynomial Contrast *p*-Values
Linear	0.562
Quadratic	0.009
Cubic	0.768

^1^ MT: diet containing mycotoxins; MT + 0.025% YCWE; MT + 0.05% YCWE; MT + 0.1% YCWE; MT + 0.2% YCWE; and MT + 0.4% YCWE.

**Table 9:** Feed formulation and nutritional composition of basal diets for starter (day 0 to 21), grower (day 21 to 35) and finisher (day 35 to 42) phases, as-fed basis.

Item	Starter	Grower	Finisher
Ingredient, %			
Corn, yellow	43.98	48.31	49.40
Distillers dried grains with solubles	25.00	25.00	25.00
Soybean meal dehulled, solvent	22.47	17.75	16.21
Fat, vegetable	4.34	5.04	5.79
Dicalcium phosphate	1.99	2.03	1.92
Calcium carbonate	1.06	0.83	0.80
L-Lysine	0.39	0.38	0.31
Methionine-MHA	0.29	0.23	0.20
Salt (as NaCl)	0.25	0.25	0.25
L-Threonine 98.5	0.08	0.06	0.00
Trace mineral ^1^	0.08	0.08	0.08
Vitamin premix ^2^	0.07	0.05	0.05
Calculated Nutrient analysis			
Dry matter (%)	88.54	88.53	88.57
Protein, crude (%)	22.0	20.0	19.2
Fat, crude (%)	7.24	8.04	8.80
Metabolizable energy (kcal/kg)	3035	3120	3180
Calcium (%)	0.9	0.8	0.76
Phosphorus, total (%)	0.83	0.82	0.79
Phosphorus, available (%)	0.40	0.40	0.38
Lysine (%)	1.33	1.19	1.09
Methionine (%)	0.63	0.55	0.51
Tryptophan (%)	0.24	0.21	0.20

^1^ Trace mineral mix provided the following (per kg of diet): manganese (MnSO_4_•H_2_O), 60 mg; iron (FeSO_4_•7H_2_O), 30 mg; zinc (ZnO), 50 mg; copper (CuSO_4_•5H_2_O), 5 mg; iodine (ethylene diamine dihydroiodide), 0.15 mg; selenium (NaSe0_3_), 0.3 mg. ^2^ Vitamin premix: vitamin mix provided the following (per kg of diet): vitamin A, 8818 IU; vitamin D3, 2480 IU; 25-hydroxyvitamin D3, 69 µg; vitamin E, 35 IU; vitamin B_12_ (cobalamin),15.5 µg; biotin, 0.17 mg; menadione, 1.98 mg; thiamine, 1.87 mg; riboflavin, 7.7 mg; d-Panthothenic acid, 13.23 mg; vitamin B6, 3.3 mg; niacin, 44.1 mg; folic acid, 1.1 mg.
